# Reversal of EGFR inhibitors’ resistance by co-delivering EGFR and integrin αvβ3 inhibitors with nanoparticles in non-small cell lung cancer

**DOI:** 10.1042/BSR20181259

**Published:** 2019-08-28

**Authors:** Fei He, Yanzhong Wang, Wanru Cai, Minjing Li, Lei Dong

**Affiliations:** 1 Department of Pneumology, Hangzhou Red Cross Hospital/Zhejiang Chinese Medicine and Western Medicine Integrated Hospital, 208 East Huancheng Road, Hangzhou 310003, Zhejiang, China; 2 Department of Clinical Laboratory, Sir Run Run Shaw Hospital, Zhejiang University School of Medicine, Hangzhou 310016, Zhejiang, China; 3 Department of Pneumology, The Second Affiliated Hospital of Zhejiang Chinese Medical University, 318 Chaowang Road, Hangzhou 310005, Zhejiang, China

**Keywords:** drug resistance, EGFR, integrin αvβ3, nanoparticles

## Abstract

**Purpose:** Tumor cells, with drug resistance, are associated with failed treatment and poor prognosis. Our aim was to explore potential strategy to overcome the epidermal growth factor receptor (EGFR) inhibitors resistance in non-small cell lung cancer (NSCLC).

**Materials and methods:** Flow cytometry was used to examine and sort cells. Using MTT assay, we detected the cell viability under different conditions. Using RT-qPCR and Western blot, we determined the targeted gene expression in mRNA and protein levels. The morphology of the prepared nanoparticles was pictured by transmission electron microscopy. We also performed immunohistochemistry (IHC) and immunofluorescence (IF) to detect the proteins expression. Subcutaneous cancer models in nude mice were constructed to evaluate the anti-cancer effects *in vivo*.

**Results**: Here, we observed enhanced expression of integrin αvβ3 in tumor tissues from EGFR inhibitors resistant patients. Also, integrin αvβ3-positive NSCLC cells revealed significant EGFR inhibitors resistance, resulting from the activation of Galectin-3/KRAS/RalB/TBK1/NF-κB signaling pathway. Co-encapsulating integrin αvβ3 inhibitor and EGFR inhibitor further improved the drug delivery system, leading to superior anti-cancer effects and reduced systemic toxicity.

**Conclusion:** Our results demonstrated that co-encapsulation of erlotinib and cilengitide by MPEG-PLA (Erlo+Cilen/PP) nanoparticles revealed enhanced tumor suppression along with reduced organ damages, providing an innovative approach for NSCLC treatment.

## Introduction

Lung cancer is the most common malignance with a high lethality worldwide [1], two main types of which are small-cell lung cancer (SCLC) and non-small cell lung cancer (NSCLC). And approximately 85–90% of lung cancers are diagnosed as NSCLC [2]. Due to the clinical advances of early diagnoses and the application of superior chemotherapeutic agents or molecule-targeted drugs, many lung cancer patients show improved prognosis and prolonged survival time after standard chemotherapy. However, high percentage of patients eventually develops drug resistance after treatment, resulting in the dismal prognosis [3]. Unfortunately, the mechanisms of drug resistance development remain unclear in NSCLC, especially the epidermal growth factor receptor (EGFR) inhibitors resistance [4]. Therefore, innovative therapeutic methods to reverse the EGFR inhibitors resistance and enhance the anti-cancer effect of NSCLC are highly desirable.

Integrins, which consist of α subunit and β subunit, are heterodimeric adhesion receptors which are associated with the cellular adhesion to the extracellular matrix and signal transduction. By now, 18 α subunits and 8 β subunits of integrins have been identified in humans, which combine to form 24 different integrin heterodimers [5]. Accumulating evidence indicates that the abnormal expression of integrins is associated with various tumor progressions, including tumor initiation [6], sustained tumor growth, distant metastasis [7] and drug resistance development [8]. Recent studies have demonstrated that some integrins, such as integrin αvβ3 and α2β1, are crucial for the maintenance of stemness in cancer cells [9]. Furthermore, integrin αvβ1 has been proved to be capable of triggering cell migration and cancer metastasis in various tumors [10]. Even the relationship between integrins and drug resistance development have been widely studied, the specific mechanisms of the drug resistance induced by integrins is still unclear. Revealing the underlying mechanism may lay an important basis to explore new targets for integrins to obstruct tumor growth in clinical cancer therapy.

In our studies, enhanced expression of integrin αvβ3 was observed in EGFR-resistant NSCLC samples. Additionally, our results revealed that the Galectin-3/KRAS/RalB/TBK1/NF-κB signaling pathway was activated by integrin αvβ3, leading to the EGFR-associated drugs resistance in NSCLC. Concomitantly, the application of integrin αvβ3 inhibitor, cilengitide (Cilen), could efficiently reverse the drug resistance. To further enhance the anti-cancer effects and avoid the side effects, we developed the MPEG-PLA nanoparticles to co-encapsulate Cilen and EGFR inhibitors. Compared with traditional drug delivery system, such as gold nanoparticles [11] or liposomes delivery systems [12], the MPEG-PLA nanoparticles reveal high drug loading (DL) and encapsulation efficiency (EE). Additionally, the biodegradable micelles enable the safety of drugs delivery system. More importantly, the Cilen and EGFR drugs loaded MPEG-PLA nanoparticles also reveal superior tumor growth inhibition along with reduced systemic toxicity, which provides a potential strategy in clinical EGFR-resistant NSCLC treatment.

## Materials and methods

### Cell lines and reagents

A549 (human NSCLC cell line), NCI-H1975 (human NSCLC cell line) and Lewis (mouse NSCLC cell line) cells were purchased from the Cell Bank of Chinese Academy of Sciences (Shanghai, China). All cell lines were cultured in RPMI-1640 (Invitrogen, CA, U.S.A.) supplemented with 10% fetal bovine serum (Gibco, CA, U.S.A.), penicillin (100 U/ml) and streptomycin (0.1 mg/ml). Doxorubicin (DOX), paclitaxel (PTX), gemcitabine (GEM), erlotinib (Erlo) [13,14], gefitinib (Gefi), lapatinib (Lapa) and Cilen [15] were purchased from Selleck (Houston, U.S.A.). MPEG-PLA (MPEG:PLA molar ratio = 50:50, molecular weight = 4000 g/mol) were purchased from Daigang (Jinan, China). Glutamic pyruvic transaminase (GPT) assay kit, glutamic oxaloacetic transaminase (GOT) assay kit and creatinine (CRE) assay kit were purchased from Solarbio (Beijing, China).

### Immunohistochemistry and immunofluorescence

NSCLC samples were obtained by fiberoptic bronchoscopy at The Second Affiliated Hospital of Zhejiang Chinese Medical University and were kept in 4% paraformaldehyde. According to the clinical data, samples were divided into newly diagnosed and EGFR resistance. All samples were confirmed as NSCLC by a pathologist expert. All subjects gave written informed consent. Ethical approval was obtained from the Committee of the Second Affiliated Hospital of Zhejiang Chinese Medical University and all researchers have been carried out in accordance with the World Medical Association Declaration of Helsinki. After fixation, the samples were processed, embedded in paraffin, and sectioned at 4 μm for further study. Antigen retrieval was done using citric acid and sodium citrate in a microwave oven (Media, China). Then the sections were incubated with integrin αvβ3 (Abcam, ab119992, 1:500, U.K.) at 4°C overnight, followed by signal amplification using an ABC HRP Kit (Thermo, U.S.A.) and counter-staining with Hematoxylin, dehydration with series of graded ethanol and cleaned with xylene. Microscope (Leica, Germany) was used to visualize the sections. For cell immunofluorescence (IF), samples were blocked in 5% bovine serum albumin in PBS for 1 h, NF-κB (CST, 8242, 1:400, U.S.A.) were incubated at 4°C overnight, followed by signal amplification using TSA Kit (PerkinElmer, U.S.A.). An Olympus confocal microscope (Japan) was used to visualize.

### Flow cytometry

Cells were collected in PBS and stained with anti-human integrin αvβ3 PE (1:100, Abcam, ab7166, Cambridge, U.K.) at 4°C for 30 min. To examine the percentage of integrin αvβ3 in patients’ samples, 1 mg/ml collagenase (Sigma–Aldrich, San Francisco, U.S.A.), 2 units/ml hyaluronidase (Sigma–Aldrich, San Francisco, U.S.A.), and 0.1 mg/ml DNase (Sigma–Aldrich, San Francisco, U.S.A.) were used to digest the tissues into single cells, the anti-human CD45 APC (1:100, eBioscience, CA, U.S.A.) and anti-human integrin αvβ3 PE (1:100, Abcam, Cambridge, U.K.) were stained at 4°C for 30 min. After washing, the data were collected by BD Canto II (BD Biosciences, NY, U.S.A.). 7-AAD was used to exclude the dead cells. IgG (1:1000, Abcam, Cambridge, U.K.) was used as the negative control. For integrin αvβ3^+^ cell sorting, BD FACSARIA III was used to sort PE positive cells.

### Cell viability assay

Cell viability was determined by MTT assay. Briefly, 3000 cells were seeded into 96-well culture plates. After 12 h, cells were treated with various concentrations of drugs or nanoparticles. After 48 h, cell growth was measured after addition of 10 μl of 0.5 mg/ml MTT solution. After 4 h of incubation at 37°C, the medium was replaced with 100 μl dimethyl sulfoxide and vortexed for 10 min. Absorbance was measured at 570 nm by a microplate reader (Bio-Rad). Each experiment was performed for at least three times.

### Preparation of Erlo+Cilen/PP

Erlo+Cilen loaded MPEG-PLA nanoparticles preparation: 95 mg MPEG-PLA, 1 mg Erlo (Lapa or Gefi) and 5 mg Cilen were co-dissolved in 3 ml dichloromethane, followed with evaporation under reduced pressure in a rotary evaporator at 60°C. Then 500 ml PBS (pH = 7.4) was used to rehydrate the film and allow the self-assembly of Erlo+Cilen loaded MPEG-PLA nanoparticles.

DL and EE of co-delivery PG NPs were calculated from the following formulas:

$$\text{Drug loading}=\frac{\text{Weight of drug in nanoparticles}}{\text{Weight of nanoparticles}}\times 100\%$$

$$\text{Encapsulation efficiency}=\frac{\text{Weight of drug in nanoparticles}}{\text{Weight of drug total used}}\times 100\%$$

### Particle size analysis

The particle size of Erlo+Cilen/PP NPs was detected by Malvern Nano ZS90 (Malvern Instruments, Malvern, U.K.). The measuring progress was performed under the temperature of 25°C. All results were performed for at least three independent experiments.

### Morphology study

The morphology of the prepared nanoparticles was pictured by transmission electron microscope (Hitachi, Tokyo, Japan). Briefly, the nanoparticles were diluted by distilled water and then placed on a copper grid covered with nitrocellulose. Samples were negatively stained with phosphotungstic acid and dried at room temperature. Each experiment was performed for at least three independent times.

### Drugs release study

*In vitro* drug release behaviors of free Cilen, free Erlo and Erlo+Cilen loaded nanoparticles were detected by a dialysis method. Briefly, 2.5 ml Cilen or Erlo in PBS, or Erlo+Cilen/PP nanoparticles were placed in dialysis bags (molecular weight cutoff, 3.5 kDa). The dialysis bags were incubated in 25 ml PBS containing 2.5 ml fetal calf serum (37°C and pH = 5.0 or 7.4) in a 50-ml centrifugal tube at 100 rpm. At predetermined time points, 2 ml of release medium was collected for the drug release analysis. The amount of released drugs was quantified by high-performance liquid chromatography (HPLC, Waters 2695; Waters Corporation, U.S.A.). For Erlo, an octadecylsilyl silica gel column (TSK-gel, 5 mm; 4.6 × 150 mm; Tosoh, Tokyo, Japan) was used. The column was maintained at room temperature. The detection wavelength was 316 nm. The mobile phase solvent consisting of acetonitrile, methanol, water and trifluoroacetic acid (26:26:48:0.1) was pumped at a flow rate of 1.0 ml/min. The Cilen release in those samples was determined by HPLC with UV detection (Agilent 1100, Chromolith RP18e 100 × 3 mm, Merck, 220 nm, eluent A = water/formic acid (999:1), eluent B = acetonitrile/formic acid (999:1), gradient: t = 0 min 90% A, t = 0.6 min 90% A, t = 4 min 10% A, t = 5.5 min 10% A, column temperature 37°C). Each experiment was performed for three independent times.

### Animal experiments

Nude mice (6–8 weeks, female) were purchased from Beijing Vital River Laboratory Animal Technology (Beijing, China) and received a subcutaneous injection with 1 million A549 cells. One week later, mice were randomly separated into different groups and treated with Erlo (50 mg/kg, daily, tail vein) [16], Gefi (200 mg/kg daily, tail vein) [17], Lapa (100 mg/kg, daily, tail vein) [18], Cilen (200 mg/kg, daily, tail vein), Erlo+Cilen/PP (50/200 mg/kg), Gefi-Cilen/PP (200/200 mg/kg), Lapa-Cilen/PP (100/200 mg/kg). Tumor volume was recorded every 3 days by length and width, meanwhile the survival of tumor-bearing mice was observed everyday. For systemic toxicity analyses, normal mice received Erlo, Cilen, Erlo+Cilen/PP treatment and the body weight was recorded every 3 days. After 1 week, mice were killed and the CRE, GPT and GOT in blood were detected. All animal studies were approved by the Animal Care and Use Committee of The Second Affiliated Hospital of Zhejiang Chinese Medical University.

### Western blot

Samples were solubilized with an equal volume of loading buffer (125 mM Tris/HCl, pH 6.8, 4% sodium dodecyl sulfate, 20% glycerol, 0.05% Bromophenol Blue, 5% β-mercaptoethanol) and were boiled for 10 min, then samples were separated by SDS/PAGE, followed by transferring to PVDF membranes and detecting by immunoblotting with primary antibodies against human integrin αvβ3 (Abcam, ab119992, 1:500, Cambridge, U.K.), Galectin-3 (CST, 87985, 1:1000, Boston, U.S.A.), KRAS (Abcam, ab180772, 1:200, Cambridge, U.K.), RalB (CST, 3523, 1:1000, Boston, U.S.A.), TBK1 (CST, 3504, 1:1000, Boston, U.S.A.), p-TBK1 (CST, 5483, 1:1000, Boston, U.S.A.), respectively, at 4°C overnight. Then HRP–conjugated secondary antibody (CST, Boston, U.S.A.) was incubated for 1 h at room temperature, and visualized by using ECL detection kit (CST, Boston, U.S.A.). β-actin (CST, 58169, 1:1000, Boston, U.S.A.) was used as an internal control.

### Statistical analysis

Results were presented as mean ± SEM and statistical significance was examined by an unpaired Student’s *t* test by the GraphPad 6.0 software. *P*-value <0.05 was considered as statistically significant.

## Results

### Integrin αvβ3^+^ cells show EGFR inhibitors resistance in NSCLC

**Figure 1:**
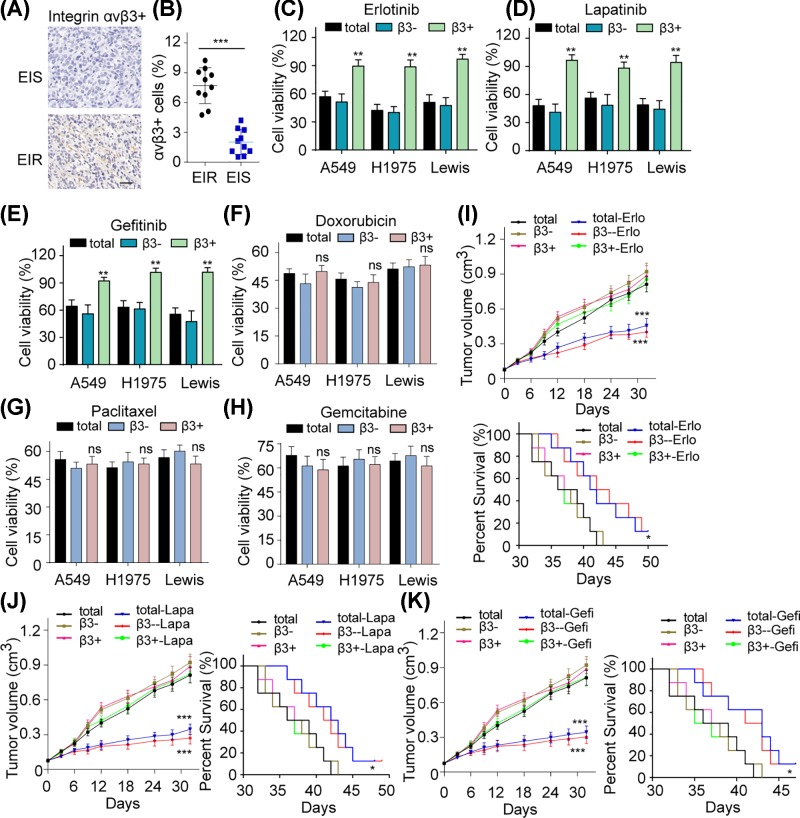
Integrin αvβ3^+^ cells show EGFR inhibitors resistance in NSCLC (**A**) The expression of integrin αvβ3^+^ tumor cells in EGFR inhibitor drugs-resistant (EIR) and -sensitive tumor (EIS) tissues obtained from NSCLC patients via using immunohistochemistry (IHC) (bar, 50 μm). (**B**) The percentage of integrin αvβ3^+^ tumor cells in EGFR inhibitor drugs-resistant and -sensitive tumor tissues obtained from NSCLC patients by using flow cytometry. (**C**) Cell viability of total, integrin αvβ3^−^ and αvβ3^+^ separated from A549, NCI-H1975 (H1975) and Lewis cells after treated with Erlo (10 μM) for 48 h. (**D**) Cell viability of total, integrin αvβ3^−^ and αvβ3^+^ from A549, NCI-H1975 and Lewis cells after treating with Lapa (10 μM) for 48 h. (**E**) Cell viability of total, integrin αvβ3^−^ and αvβ3^+^ from A549, NCI-H1975 and Lewis cells after treated with Gefi (3 μM for A549 and 10 μM for NCI-H1975/Lewis, respectively) for 48 h. (**F**) Cell viability of total, integrin αvβ3^−^ and αvβ3^+^ from A549, NCI-H1975 and Lewis cells after treated with doxorubicin (10 nM) for 48 h. (**G**) Cell viability of total, integrin αvβ3^−^ and αvβ3^+^ from A549, NCI-H1975 and Lewis cells after treated with PTX (30 nM for A549/NCI-H1975 cells and 60 nM for Lewis cells, respectively) for 48 h. (**H**) Cell viability of total, integrin αvβ3^−^ and αvβ3^+^ from A549, NCI-H1975 and Lewis cells after treated with GEM (1 μM for A549/NCI-H1975 cells and 3 μM for Lewis cells, respectively) for 48 h. (**I**) Tumor volume of nude mice bearing total, integrin αvβ3^−^ and αvβ3^+^ separated from A549 cells treated with PBS or Erlo (50 mg/kg) were measured (*n*=8) (up). Survival time of nude mice bearing total, integrin αvβ3^−^ and αvβ3^+^ from A549 cells treated with PBS or Erlo (50 mg/kg) in each group were recorded (*n*=8) (down). (**J**) Tumor volume of nude mice bearing total, integrin αvβ3^−^ and αvβ3^+^ from A549 cells treated with PBS or Lapa (100 mg/kg) were measured (*n*=8) (left). Survival time of nude mice model bearing total, integrin αvβ3^−^ and αvβ3^+^ from A549 cells treated with PBS or Lapa (100 mg/kg) in each group were recorded (*n*=8) (right). (**K**) Tumor volume of nude mice bearing total, integrin αvβ3^−^ and αvβ3^+^ separated from A549 cells treated with PBS or Gefi (200 mg/kg) were measured (*n*=8) (left). Survival time of nude mice model bearing total, integrin αvβ3^−^ and αvβ3^+^ from A549 cells treated with PBS or Gefi (200 mg/kg) in each group were recorded (*n*=8) (right). *P<0.05; **P<0.01; ***P<0.001; ns, no significant difference.

Most NSCLC patients receiving anti-EGFR therapies have benefited from the treatment, however, the development of drug resistance is accompanied with the target therapy, resulting in the poor prognosis [19]. To assess a potential strategy for eliminating EGFR resistance, the newly diagnosed and EGFR inhibitors resistant NSCLC patients’ samples were collected and analyzed in our studies. Previous reports have demonstrated that integrin family is associated with various tumor progressions [20]. And some integrin dimers, such as integrin α6β4, play a crucial role in cancer drug resistance development [21]. Intriguingly, we found that integrin αvβ3 positive cancer cells were highly enriched in EGFR-resistant samples (Figure 1A,B). This meaningful finding reminded us that integrin αvβ3 positive cells appear to be associated with the EGFR inhibitors resistance development in NSCLC patients. To further testify the relation between integrin αvβ3 and EGFR inhibitors resistance, we sorted integrin αvβ3^+^ and αvβ3^−^ cells from NSCLC cell lines (A549, NCI-H1975 and Lewis) and treated with three clinical EGFR inhibitors (Erlo, Gefi and Lapa). Compared with the αvβ3^−^ or the control groups, the integrin αvβ3^+^ cancer cells showed significant EGFR inhibitors resistance (Figure 1C–E), suggesting that the expression of integrin αvβ3 are highly correlated to the EGFR-associated drugs resistance in NSCLC. Simultaneously, it is questionable whether the integrin αvβ3^+^ cells possessed other chemotherapeutic agents resistance. We applied DOX, PTX and GEM to treat the total, integrin αvβ3^−^ and the integrin αvβ3^+^ cells, and we found that these drugs inhibited the growth of total, integrin αvβ3^−^ and integrin αvβ3^+^ cells (Figure 1F–H). Those data suggest that the integrin αvβ3^+^ subpopulation cells exhibit EGFR inhibitors resistance and remain sensitive to chemotherapeutic agents *in vitro*. To further demonstrate the EGFR-associated drugs resistance induced by integrin αvβ3 *in vivo*, the A549 subcutaneous nude mice model was constructed by the injection of total, integrin αvβ3^−^ and integrin αvβ3^+^ A549 cells. EGFR inhibitors were used to treat those tumor-bearing mice and we observed that Erlo, Gefi and Lapa restrained the tumor growth and prolonged the tumor-bearing mice survival time in total and integrin αvβ3^−^ A549 tumors while the mice bearing αvβ3^+^ A549 tumor revealed significant drug resistance (Figure 1I–K). Taking together, these findings indicate that the integrin αvβ3^+^ cells maintain the EGFR resistance in NSCLC.

### EGFR inhibitors drive integrin αvβ3^+^ cells enrichment in NSCLC

**Figure 2:**
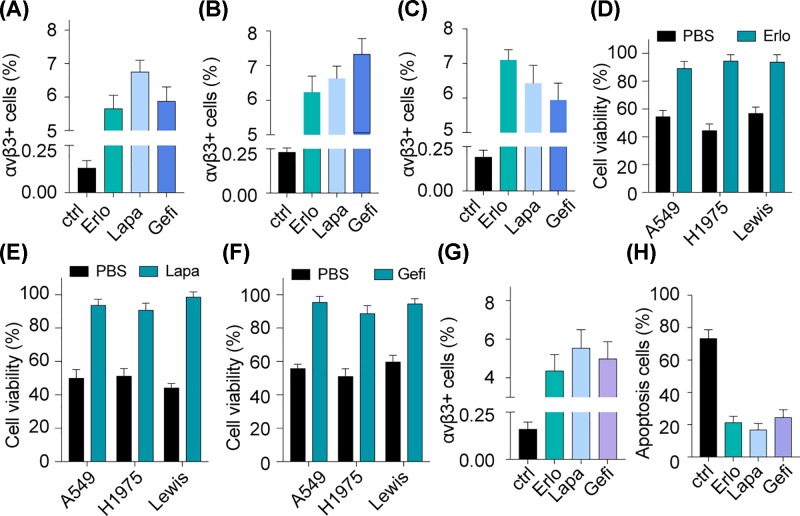
EGFR inhibitors drive integrin αvβ3^+^ cells enrichment in NSCLC (**A**) The percent of integrin αvβ3^+^ cells in A549 received PBS, Erlo (10 μM), Lapa (10 μM) or Gefi (3 μM) treatment was detected by using flow cytometry. (**B**) The percent of integrin αvβ3^+^ cells in NCI-H1975 received PBS, Erlo (10 μM), Lapa (10 μM) and Gefi (10 μM) treatment was detected by using flow cytometry. (**C**) The percent of integrin αvβ3^+^ cells in Lewis received PBS, Erlo (10 μM), Lapa (10 μM) and Gefi (10 μM) treatment was detected by using flow cytometry. (**D**) A549, NCI-H1975 and Lewis cells were pre-treated with Erlo (10 μM) for 48 h. Then the living cells were retreated by Erlo (10 μM) for another 48 h, followed by cell viability detection (*n*=3). (**E**) A549, NCI-H1975 and Lewis cells were pre-treated with Lapa (10 μM) for 48 h. Then the living cells were retreated by Lapa (10 μM) for another 48 h, followed by cell viability detection (*n*=3). (**F**) A549, NCI-H1975 and Lewis cells were pre-treated with Gefi (3/10/10 μM) for 48 h. Then the living cells were retreated by Gefi (3/10/10 μM) for another 48 h, followed by cell viability detection (*n*=3). (**G**) The nude mice bearing A549 cells were treated with PBS, Erlo (50 mg/kg), Lapa (100 mg/kg) or Gefi (50 mg/kg) for 3 days, then the percent of integrin αvβ3^+^ cells in tumor tissue were detected by using flow cytometry (*n*=8). (**H**) The nude mice bearing A549 cells were treated with PBS or Erlo (50 mg/kg) for 3 days, then the tumor tissues were excised and treated with PBS, Erlo (10 μM), Lapa (10 μM) or Gefi (10 μM) respectively for 48 h *in vitro*. Then the apoptotic rate was detected by using flow cytometry (*n*=5).

Integrin αvβ3 serves as the membrane receptors, which is constitutively expressed on many cells in tissues, including the NSCLC cells. However, EGFR is a transmembrane protein as well and overexpressed in a wide variety of tumor cells [22,23]. Here, we wondered that how integrin αvβ3 induces the EGFR inhibitors resistance in NSCLC. Thus, we made A549, NCI-H1975 and Lewis cell lines exposure to EGFR inhibitors (Erlo, Gefi and Lapa) directly and then detected the expression of integrin αvβ3 in those cells. Intriguingly, the integrin αvβ3^+^ cells percentages were increased after treatment of those inhibitors respectively (Figure 2A–C), which corroborated the clinical findings that integrin αvβ3^+^ cells accumulated in EGFR-resistant patients. Next, we collected the remnant living tumor cells after inhibitors treatment and then treated them with Erlo, Gefi and Lapa, respectively. Expectedly, those EGFR inhibitors were not able to suppress the three NSCLC cells lines again, indicating the EGFR inhibitors resistance development in those cells (Figure 2D–F). Those findings suggest that EGFR inhibitors suppressed the growth of αvβ3^−^ cancer cells while having no inhibitory effects on those αvβ3^+^ cancer cells, which concentrated the integrin αvβ3^+^ cells to induce EGFR inhibitors resistance *in vitro*. Meanwhile, we used EGFR inhibitors to treat A549 bearing nude mice and sort the residual tumor cells. We noted that the number of integrin αvβ3^+^ cells increased after EGFR inhibitors treatment *in vivo* (Figure 2G). Similar to the result *in vitro*, EGFR inhibitors failed to induce those residual tumor cells apoptosis (Figure 2H). Together, the above data revealed that the treatment of EGFR inhibitors could suppress the αvβ3^−^ tumor cells proliferation and drive the integrin αvβ3^+^ cells accumulation, leading to EGFR inhibitors resistance in NSCLC.

### Integrin αvβ3^+^ cells induce EGFR resistance through Galectin-3/KRAS/RalB/TBK1/NF-κB signaling pathway

**Figure 3:**
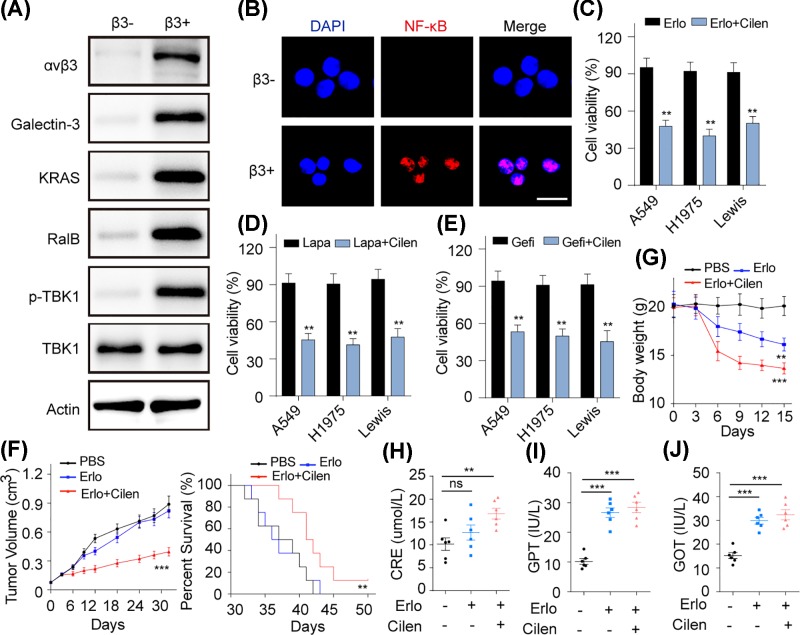
Integrin αvβ3^+^ cells induce EGFR resistance through Galectin-3/KRAS/RalB/TBK1/NF-κB signaling pathway (**A**) The expressions of integrin αvβ3, Glatin-3, KRAS, RalB, p-TBK1, TBK1 and actin in integrin αvβ3^−^ and αvβ3^+^ A549 cells were detected by using Western blot. (**B**) The distribution of NF-κB in integrin αvβ3^−^ and αvβ3^+^ A549 cells were examined by using confocal (bar, 50 μM). (**C**) Cell viability of A549, NCI-H1975 and Lewis cells treated with Erlo (10 μM) with or without Cilen (5 μM) for 48 h. (**D**) Cell viability of A549, NCI-H1975 and Lewis cells treated Lapa (10 μM) with or without Cilen (5 μM) for 48 h. (**E**) Cell viability of A549, NCI-H1975 and Lewis cells treated Gefi (3/10/10 μM) with or without Cilen (5 μM) for 48 h. (**F**) Tumor volume of nude mice bearing integrin αvβ3^+^ A549 cells treated with PBS, Erlo (50 mg/kg) or Erlo (50 mg/kg) combining with Cilen (200 mg/kg) were measured (*n*=8) (left). Survival time of nude mice bearing αvβ3^+^ A549 cells treated with PBS, Erlo (50 mg/kg) with Cilen (200 mg/kg) were recorded (*n*=8) (right). (**G**) The body weight of C57 mice receiving PBS, Erlo (50 mg/kg) and Erlo (50 mg/kg) + Cilen (200 mg/kg) (*n*=6) treatment was measured. (**H**) The concentrations of CRE were detected in C57 mice receiving PBS, Erlo (50 mg/kg) and Erlo (50 mg/kg) + Cilen (200 mg/kg). (**I**) The levels of GPT were detected in C57 mice treated with PBS, Erlo (50 mg/kg) and Erlo (50 mg/kg) + Cilen (200 mg/kg). (**J**) The levels of GOT were detected in in C57 mice received PBS, Erlo (50 mg/kg) and Erlo (50 mg/kg) + Cilen (200 mg/kg). **P<0.01; ***P<0.001, ns, not statistically significant.

Next, we wondered how the integrin αvβ3^+^ cells cause the EGFR resistance. Studies have reported that Galectin-3 was an interlinkage between integrin αvβ3 and KRAS [24,25]. More importantly, these molecules were considered to be associated with the tumor stemness, metastasis and drug resistance. Therefore, we considered whether Galectin-3/KRAS could be activated by integrin αvβ3. And we found that both Galectin-3 and KRAS were activated in integrin αvβ3^+^ cells comparing with integrin αvβ3^−^ cells (Figure 3A). Also, as the classic downstream molecule of KRAS, RalB was activated and TBK1 was phosphorylated (Figure 3A), which is in line with the previous studies [26]. Increasing evidence indicates that TBK1 could regulate the NF-κB signals, which is associated with EGFR-associated drug resistance acquisition in tumor cells. In our study, we observed that integrin αvβ3 activation triggered NF-κB into nucleus (Figure 3B). Those data demonstrate that Galectin-3/KRAS/RalB/TBK1/NF-κB signaling pathway assists the integrin αvβ3^+^ cells to hold EGFR inhibitors resistance. Then, we were thinking about how to conquer the EGFR resistance. In general, targeting the membrane protein should be more specific and effective for cancer therapy. Thus, integrin αvβ3 might be the ideal target. Cilen, the specific inhibitor of integrin αvβ3, is an ideal agent to reverse the drug resistance induced by integrin αvβ3. Herein, we combined Cilen with EGFR inhibitors to treat integrin αvβ3^+^ A549, NCI-H1975 and Lewis cells. As predicted, the addition of Cilen significant enhanced the cytotoxicity of EGFR inhibitors to those integrin αvβ3^+^ tumor cells (Figure 3C–E). Further, combination of Cilen and Erlo restricted the integrin αvβ3^+^ A549 tumor growth and prolonged the survival time of those tumor-bearing mice (Figure 3F). Except for the anti-cancer effects, the potential toxicity of agents is a crucial index for clinical cancer therapy. Unfortunately, we found that this combing strategy decreased the normal mice body weight seriously (Figure 3G) and increased the CRE, GPT and GOT in blood comparing with PBS or Erlo-treated groups, indicating the potential toxicity for liver and kidney (Figure 3H–J). Together, those findings suggest that the integrin αvβ3^+^ cells induce EGFR resistance through Galectin-3/KRAS/RalB/TBK1/NF-κB signaling pathway and targeting integrin αvβ3 with Cilen overcomes EGFR resistance *in vitro* and *in vivo*. However, direct combination of Cilen and EGFR inhibitors might result in adverse reactions *in vivo*, which limits the potential application in clinical NSCLC treatment. Therefore, improved drugs delivery system might be designed for preferable curative effects.

### Preparation and characterization of Erlo+Cilen/MPEG-PLA NPs

**Figure 4:**
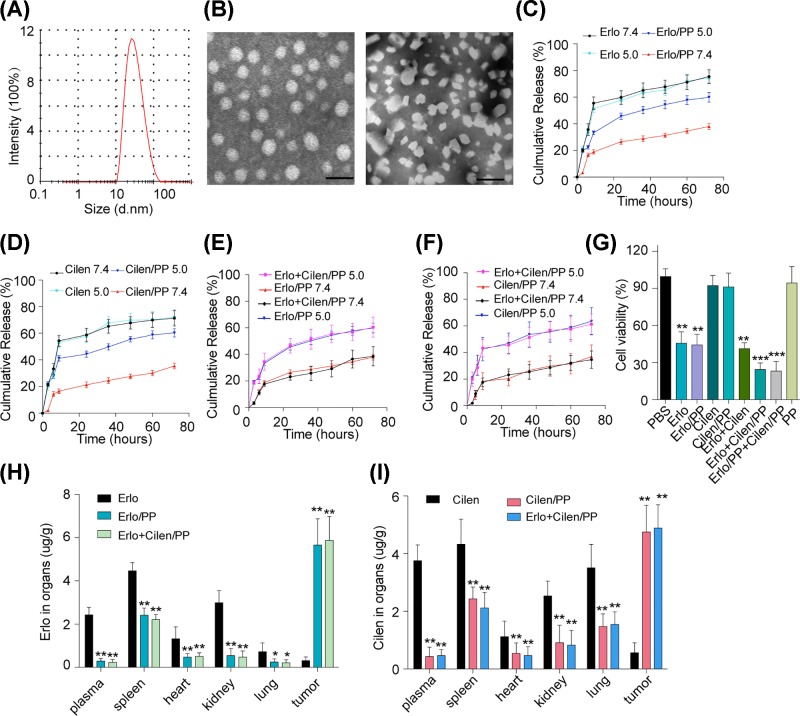
Preparation and characterization of Erlo+Cilen/MPEG-PLA NPs (**A**) The size distribution spectrum of MPEG-PLA nanoparticles was examined. (**B**) The image of MPEG-PLA nanoparticles with Erlo and Cilen (left) and after complete release the drug molecules (right) were shown by using TEM (bar, 40 nm). (**C**) Drug release of free Erlo and Erlo/PP (MPEG-PLA) under different pH conditions (pH 5.0 and 7.4) was measured (*n*=3). (**D**) Drug release of free Cilen and Cilen/PP (MPEG-PLA) under different pH conditions (pH 5.0 and 7.4) was measured (*n*=3). (**E**) Drug release of Erlo in Erlo/PP or Erlo+Cilen/PP under different pH conditions (pH 5.0 and 7.4) was measured (*n*=3). (**F**) Drug release of Cilen in Cilen/PP or Erlo+Cilen/PP under different pH conditions (pH 5.0 and 7.4) was measured (*n*=3). (**G**) The cell viability of A549 cells after treating with PBS, PP, Erlo, Erlo/PP, Cilen, Cilen/PP, Erlo+Cilen, Erlo/PP+Cilen/PP or Erlo+Cilen/PP for 48 h was detected by using MTT. (**H**) The nude mice bearing A549 cells were treated with Erlo (50 mg/kg), Erlo alone encapsulated or co-encapsulating with Cilen by MPEG-PLA (Erlo concentration 50 mg/kg) every 2 days for 10 days. Then the mice were killed to obtain blood, tumor tissues and organs. HPLC was used to detect the concentration of Erlo in the samples (*n*=6). (**I**) The nude mice bearing A549 cells were treated with Cilen (200 mg/kg), Cilen alone encapsulated or co-encapsulating with Erlo by MPEG-PLA (Cilen concentration 200 mg/kg) every 2 days for 10 days. Then the mice were killed to obtain blood, tumor tissues and organs. HPLC was used to detect the concentration of Cilen in the samples (*n*=6). PP was short for MPEG-PLA. **, *P*<0.01; ***, *P*<0.001; ns, not statistically significant.

Accumulating evidence implicates that successful drugs encapsulation by nanoparticles based on high polymers is capable of improving the drugs delivery system and pharmacokinetics profiles, leading to enhanced anti-cancer effects and reduced systemic toxicity, which is induced by the enhanced permeability and retention (EPR) effects and selective pH release [27]. MPEG-PLA nanoparticles, composed of polyethylene glycol and polylactic acid, which has been proved to be favorable drugs carriers in tumor treatment due its safety to organism and the low clearance rate in peripheral blood [28]. Here, we designed MPEG-PLA nanoparticles as a nanocarrier to co-encapsulate Erlo and Cilen to form the Erlo and Cilen co-loaded MPEG-PLA nanoparticles. When the in-feed mass ratio of Erlo/Cilen/MPEG-PLA was 1/4/95, the NPs had an EE of 97.30% (Erlo) and 94.27% (Cilen), and DL of 0.97% (Erlo) and 3.76% (Cilen). As revealed in Figure 4A, the average size of Erlo+Cilen/PP was 26 nm, which made it suitable for *in vivo* application. The transmission electron microscopy image of Erlo+Cilen/PP with drug or after complete release of drug was shown in Figure 4B. Nanoparticle-mediated cellular response is size dependent and tumor cells could uptake particles with a size below 100 nm. Our Erlo+Cilen/PP with an average particle size of 26 nm were appropriate for the drug delivery system, along with EPR effects in tumor therapy. Next, we investigated the drug release in Erlo/PP, Cilen/PP and Erlo+Cilen/PP with a dialysis method. As present in Figure 4C,D, both Erlo and/or Cilen encapsulated in MPEG-PLA nanoparticles have more drug retention in pH 7.4 (normal tissues) than free drugs, and the retained Erlo or Cilen were released from drug-coated nanoparticles in pH 5.0 (tumor microenvironment). This was very advantageous to promote the drug release of Erlo or Cilen/PP in tumor microenvironment and to facilitate the drugs aggregation in physiological tissues (pH∼7.4), resulting in selective drugs release at tumor sites to minimize the system toxicity. The same results were observed in the drug release of Erlo+Cilen loading MEPG-PLA nanoparticles (Figure 4E,F). Next, to further evaluate the cell cytotoxicity of Erlo+Cilen/PP. We used PP, Erlo/PP, Cilen/PP, Erlo/PP+Cilen/PP and Erlo+Cilen/PP to treat the integrin αvβ3^+^ A549 cells and we noted that Erlo/PP slightly decreased the cell viability. Moreover, both Erlo/PP+Cilen/PP and Erlo+Cilen/PP markedly reduced the cell viability than Erlo/PP in these cells, while PP and Cilen/PP treatment could not induce cell cytotoxicity (Figure 4G). More importantly, encapsulation of Erlo and Cilen by MPEG-PLA nanoparticles facilitated the drug accumulation in tumor site instead of distribution in normal tissues due to the EPR effects and selective release induced by pH (Figure 4H,I). The enhanced tumor cytotoxicity and selective release could efficiently enhance tumor suppression and avoid the potential kidney and liver damage induced by two agents. Moreover, co-encapsulation of the Erlo and Cilen enabled the tumor cells to uptake two agents simultaneously, which could efficiently overcome the drug resistance induced by integrin αvβ3 and ensure the cytotoxicity. Taken together, those results indicate that the encapsulation of Erlo+Cilen/PP enhances the selective drug release at tumor site and it could be expected to achieve anti-cancer effects and reduced systemic toxicity.

### Erlo+Cilen/PP reverses the EGFR resistance to enhance the tumor suppression and reduces systemic toxicity

**Figure 5:**
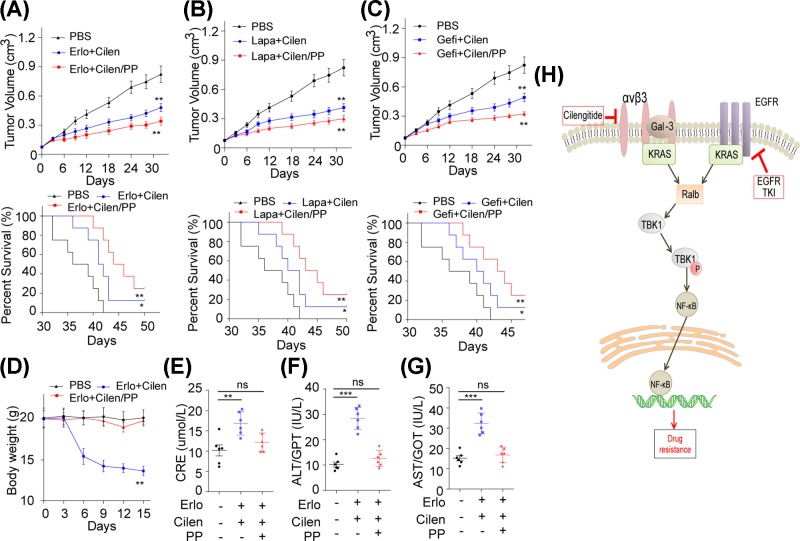
Erlo+Cilen/PP reverses the EGFR resistance to enhance the tumor suppression and reduces systemic toxicity (**A**) Tumor volume of nude mice bearing integrin αvβ3^+^ A549 cells treated with PBS, Erlo (50 mg/kg) combined with Cilen (200 mg/kg) or MPEG-PLA encapsulated Erlo (50 mg/kg) and Cilen (200 mg/kg) were measured (*n*=8) (upper). Survival time of nude mice bearing integrin αvβ3^+^ A549 cells treated with PBS, Erlo (50 mg/kg) combined with Cilen (200 mg/kg) or MPEG-PLA encapsulated Erlo (50 mg/kg) and Cilen (200 mg/kg) in each group were recorded (*n*=8) (down). (**B**) Tumor volume of nude mice bearing integrin αvβ3^+^ A549 cells treated with PBS, Lapa (100 mg/kg) combined with Cilen (200 mg/kg) or MPEG-PLA encapsulated Lapa (100 mg/kg) and Cilen (200 mg/kg) were measured (*n*=8) (upper). Survival time of nude mice bearing integrin αvβ3^+^ A549 cells treated with PBS, Lapa (100 mg/kg) combined with Cilen (200 mg/kg) or MPEG-PLA encapsulated Lapa (100 mg/kg) and Cilen (200 mg/kg) in each group were recorded (*n*=8) (down). (**C**) Tumor volume of nude mice bearing integrin αvβ3^+^ A549 cells treated with PBS, Gefi (50 mg/kg) combining with Cilen (200 mg/kg) or MPEG-PLA encapsulated Gefi (50 mg/kg) and Cilen (200 mg/kg) were measured (*n*=8) (upper). Survival time of nude mice bearing integrin αvβ3^+^ A549 cells treated with PBS, Gefi (50 mg/kg) combined with Cilen (200 mg/kg) or MPEG-PLA encapsulated Gefi (50 mg/kg) and Cilen (200 mg/kg) in each group were recorded (*n*=8) (down). (**D**) The body weight of C57 mice was measured in PBS, Erlo (50 mg/kg) + Cilen (200 mg/kg) or MPEG-PLA encapsulated Erlo (50 mg/kg) + Cilen (200 mg/kg) groups (*n*=6). (**E**) The concentrations of CRE were detected in C57 mice received PBS, Erlo (50 mg/kg) + Cilen (200 mg/kg) or MPEG-PLA encapsulating Erlo (50 mg/kg) + Cilen (200 mg/kg). (**F**) The levels of GPT were detected in C57 mice treated with PBS, Erlo (50 mg/kg) + Cilen (200 mg/kg) or MPEG-PLA encapsulating Erlo (50 mg/kg) + Cilen (200 mg/kg). (**G**) The levels of GOT were detected in C57 mice receiving PBS, Erlo (50 mg/kg) + Cilen (200 mg/kg) or MPEG-PLA encapsulating Erlo (50 mg/kg) + Cilen (200 mg/kg). (**H**) The schematic diagram of EGFR inhibitor resistance induced by integrin αvβ3 in NSCLC. *P<0.05; **P<0.01; ***P<0.001; ns, no significant difference.

To study the anti-cancer effect of EGFR inhibitors Cilen/PP in EGFR-resistant integrin αvβ3^+^ NSCLC cells, the integrin αvβ3^+^ A549 cells were inoculated in nude mice to establish the EGFR inhibitors resistant tumor model. Here, we found that Erlo+Cilen/PP efficiently restrained tumor growth and prolonged the tumor-bearing mice survival compared with the free drugs groups (Figure 5A). In addition, we also observed the same results in MPEG-PLA nanoparticles co-encapsulating Cilen and other EGFR inhibitors (Lapa and Gefi) (Figure 5B,C). Next, we wondered whether the encapsulation of nanoparticles could avoid the systemic toxicity by EPR effects and selective release. Here, we treated mice with PBS, Erlo+Cilen and Erlo+Cilen/PP every 3 days for a week. We found that Erlo+Cilen/PP did not have significant effects on body weight compared with PBS group (Figure 5D). Additionally, the application of Erlo+Cilen/PP significantly reduced the kidney and liver damages compared with the Erlo+Cilen group (Figure 5E–G), indicating that encapsulation by MPEG-PLA nanoparticles could efficiently improve the drugs delivery system to avoid system toxicity. Together, the above findings demonstrate that MPEG-PLA nanoparticles co-encapsulating EGFR inhibitors and integrin αvβ3 inhibitor Cilen could reverse the EGFR resistance along with reduced organ damages, which provides potential strategy for clinical NSCLC treatment.

## Discussion

The development of drug resistance is controlled by various factors, including the up-regulation of ATP-binding cassette (ABC) transporters protein, DNA damages repair, expression of anti-apoptotic protein such as BCL-2 expression, and activation of pro-survival pathway induced by cellular surface receptors [21,29,30]. Previous studies have demonstrated that integrins participated in various tumor progressions via the activation of several pro-survival signaling pathways in tumor cells [7]. In our studies, we proved that integrin αvβ3 induced the EGFR drugs resistance via the activation of Galectin-3/KRAS/RalB/TBK1/NF-κB signaling pathway. And co-encapsulation of EGFR agents and integrin αvβ3 inhibitors efficiently reverse the drug resistance along with reduced systemic toxicity.

It has been demonstrated that integrins family served as the cancer stem cells markers, which regulate the cancer stemness and promote tumor progressions in a wide range of tumors [10,31]. Our study further expounded the relationship between the EGFR drugs resistance and the integrin αvβ3 in NSCLC, which discloses a more fundamental role of integrin αvβ3 in tumor growth. We further verified that integrin αvβ3 activates the galectin-3 and KRAS, leading to the recruitment and phosphorylation of RalB. The phosphorylated RalB further facilitated the up-regulation of TBK1, resulting in the activation of NF-κB signaling pathway and development of EGFR drugs resistance (Figure 5H). Our results explain the role of RAS family and prove that RalB and TKB1 participate in the activation of pro-survival signaling pathway in lung cancer. More importantly, the combination of EGFR drugs and integrin αvβ3 inhibitors revealed superior anti-cancer effects, which provides a feasible approach in clinical lung cancer treatment (Figure 5H).

Unfortunately, traditional anti-EGFR drugs reveal severe side effects, such as tetter, diarrhea, renal toxicity and so on [32]. To further improve the drugs delivery system and pharmacokinetics profiles, we co-encapsulated the EGFR drugs and integrin αvβ3 inhibitors to achieve enhanced anti-cancer effects and reduced systemic toxicity. However, drugs encapsulation based on liposomes [33–35] is also accompanied with severe system toxicity. The gold nanoparticles or some polymer nanoparticles, such as PEG, are limited by their drugs loading limitation or potential safety risks due to their low biocompatibility. Compared with traditional drug delivery systems, the MPEG-PLA nanoparticles co-encapsulating EGFR drugs and integrin αvβ3 inhibitor had an average size of 25 nm with a high stability and low clearance rate, which was preferred by tumor tissues due to the EPR effects and selected release of drugs induced by environment pH. Those characters facilitate the drugs aggregation in tumor tissues and reduce the drug distribution in organs *in vivo*. According to previous reports and the optimal proportion of Cilen, Erlo (50 mg/kg) [16], Gefi (200 mg/kg) [17], Lapa (100 mg/kg) [18], Cilen (200 mg/kg) were co-encapsulated into MPEG-PLA nanoparticles for anticancer effects analysis *in vivo*. We co-encapsulated the chemotherapeutic agents and integrin αvβ3 inhibitor, and found that the co-encapsulation of EGFR drugs and integrin αvβ3 inhibitor by MPEG-PLA nanoparticles enables the tumor cells to uptake the EGFR drugs and integrin αvβ3 inhibitor simultaneously, which efficiently reverses the drug resistance and enhanced the cytotoxicity to tumor cells. Compared with previous reports, the similar dose of chemotherapeutic agents also induced superior anti-cancer effects after co-encapsulation with αvβ3 inhibitor, along with reduced systemic toxicity. Apart from the advantages of improved drugs delivery systems based on nanoparticles, the MPEG-PLA nanoparticles composed of degradable polyethylene glycol and polylactic acid, which were approved by the U.S. Food and Drug Administration, ensuring the safety as drugs delivery carriers compared with other nanoparticles [36]. Moreover, the nanoparticle based on co-delivery of EGFR drugs and integrin αvβ3 inhibitors also reveals great potential for use in the other chemotherapeutic agents, which provide a favorable platform for combined drugs delivery in cancer therapy.

In conclusion, we defined the integrin αvβ3 as the driver of EGFR drugs resistance via the activation of RalB/TBK1/NF-κB signaling pathway. We also revealed that co-delivery of integrin αvβ3 inhibitors and EGFR agents by MPEG-PLA nanoparticles could efficiently reverse the drug resistance induced by integrin αvβ3 and provide a promising strategy in the treatment of NSCLC.

## Abbreviations

7-AAD: 7-amino actinomycin D
Bcl-2: B cell lymphoma/leukemia-2
Cilen: cilengitide
CRE: creatinine
CST: cell signaling technology
DL: drug loading
DOX: doxorubicin
EE: encapsulation efficiency
EGFR: epidermal growth factor receptor
EPR: enhanced permeability and retention
Erlo: erlotinib
Gefi: gefitinib
GEM: gemcitabine
GOT: glutamic oxaloacetic transaminase
GPT: glutamic pyruvic transaminase
HPLC: high-performance liquid chromatography
HRP: Horseradish Peroxidase
KRAS/TBK1: kirsten rat sarcoma viral oncogene/Tankbinding kinase 1
Lapa: lapatinib
MPEG-PLA: methoxy poly (ethylene glycol)-poly (lactide)
NSCLC: non-small cell lung cancer
PTX: paclitaxel
RPMI-1640: roswell park memorial institute
RT-qPCR: reverse transcription quantitative polymerase chain reaction
